# Enantioseparation and Absolute Configuration Determination of Angular-Type Pyranocoumarins from Peucedani Radix Using Enzymatic Hydrolysis and Chiral HPLC-MS/MS Analysis

**DOI:** 10.3390/molecules17044236

**Published:** 2012-04-10

**Authors:** Yue-Lin Song, Qing-Wen Zhang, Ya-Ping Li, Ru Yan, Yi-Tao Wang

**Affiliations:** 1 State Key Laboratory of Quality Research in Chinese Medicine, Institute of Chinese Medical Sciences, University of Macau, Taipa, Macao SAR, 999078, China; 2 School of Chinese Materia Medica, Beijing University of Chinese Medicine, Beijing 100029, China

**Keywords:** angular-type pyranocoumarins, *cis*-khellactone, absolute configuration, enzymatic hydrolysis, chiral LC-MS/MS

## Abstract

Angular-type pyranocoumarins from Peucedani Radix (Chinese name: Qian-hu) have exhibited potential for use on treatment of cancer and pulmonary hypertension. Due to the existence of C-3′ and C-4′ chiral centers, compounds belonging to this chemical type commonly exist in enantiomers and/or diastereoisomers, which may elicit distinct activities during their interactions with the human body. In the present study, a new method, which combines enzymatic hydrolysis with chiral LC-MS/MS analysis, has been developed to determine the absolute configurations of these angular-type pyranocoumarins. Pyranocoumarins isolated from Qian-hu, their enantiomers, or metabolites were individually incubated with rat liver microsomes. As the common end product from enzymatic hydrolysis of all tested pyranocoumarins, *cis*-khellactone was collected and its absolute configuration was determined by comparison with (+)-*cis*-khellactone and (−)-*cis*-khellactone using chiral LC-MS/MS. The absolute configurations of all tested parent pyranocoumarins were determined by combination of LC-MS/MS, NMR and polarimetric analysis. The results revealed that the metabolite *cis*-khellactone retained the same absolute configurations of the stereogenic carbons as the respective parent compound. This method was proven to be rapid and sensitive and also has advantages in discriminating single enantiomers and mixtures of optical isomers with different ratios.

## 1. Introduction

Peucedani Radix (Chinese name: Qian-hu) originates from the dried roots of *Peucedanum praeruptorum* DUNN, and has been used in traditional medicinal practice in China for the treatment of cough with thick sputum and dyspnea, nonproductive cough and upper respiratory infections [1,2]. Modern pharmacological studies have revealed potent hypotensive, coronary dilatory, myocardial protection and antitumor effects of this medicinal herb [2,3,4,5].

**Figure 1:**
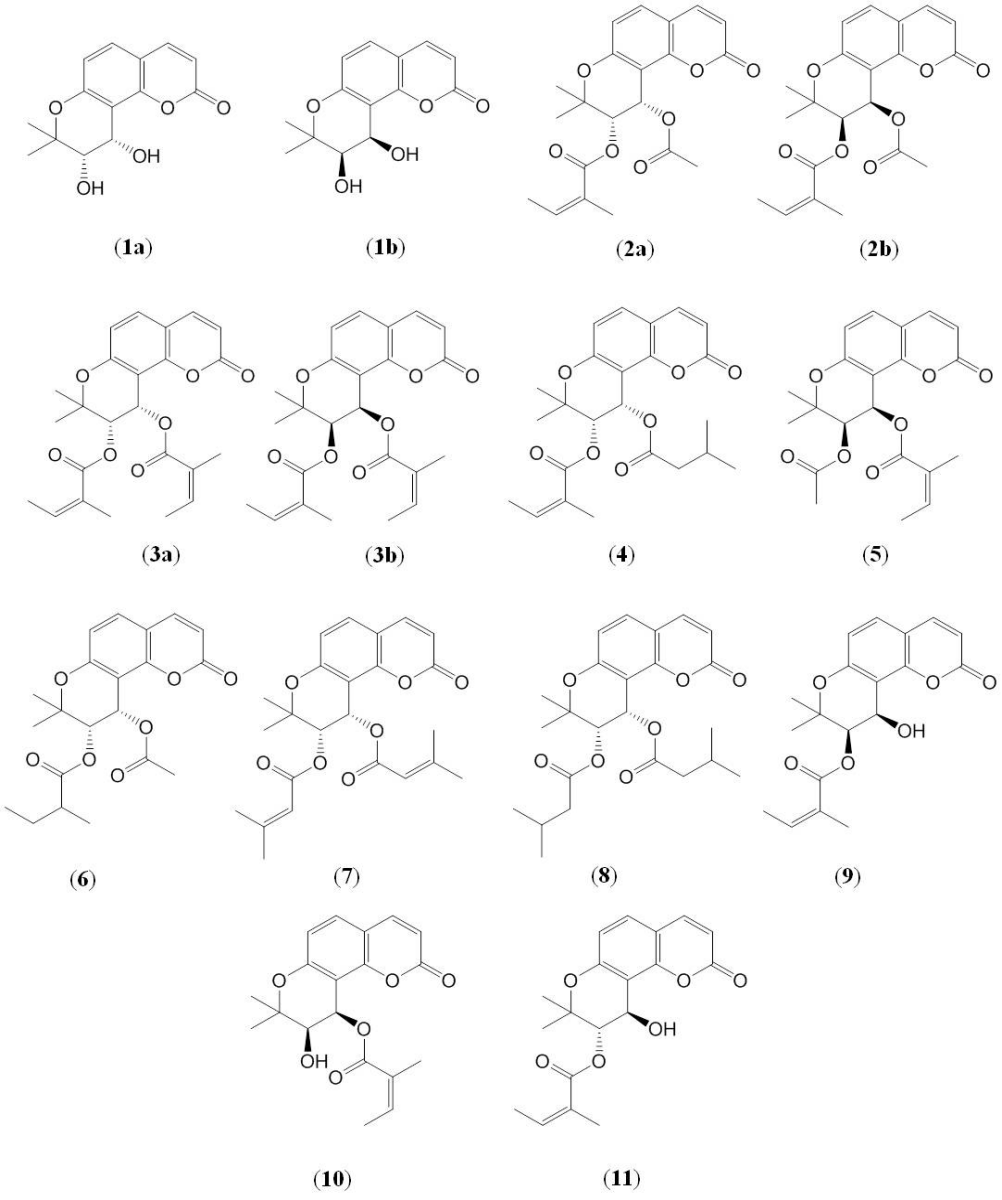
Chemical structures of pyranocoumarin compounds **1**–**11**.

Angular-type pyranocoumarins, which comprise a khellactone (**1**, Figure 1) skeleton with C-3′ and C-4′ substituents, were reported as the main bioactive constituents of Qian-hu [6,7,8,9]. In the Chinese Pharmacopeia, praeruptorin A (*cis-*3′-angeloyl-4′-acetylkhellactone, **2**) and praeruptorin B (*cis*-3′,4′-diangeloylkhellactone, **3**) were documented as the chemical markers for quality control of this herb and related products [1]. Praeruptorin A and its analogues were revealed to be cytotoxic to some cancer cells [10,11,12] and were able to induce apoptosis as well as reverse multidrug resistance (MDR) [5] by suppressing expression of the efflux transporter P-glycoprotein [11]. Furthermore, the anticancer activity of praeruptorin A has also been demonstrated in mice [12], indicating the potential of these angular-type pyranocoumarins for cancer therapy. More interestingly, the two enantiomers of praeruptorin A (**2a** and **2b**, Figure 1) initiated distinct relaxant effects on isolated rat aorta rings dependent on endothelium and nitric oxide synthesis [13], indicating the existence of stereoselectivity in pharmacological activity of praeruptorin A.

Angular-type pyranocoumarins have two stereogenic centers (C-3′ and C-4′). Enantiomers and diastereoisomers of this type of compounds naturally occur in some plants. In Qian-hu, the levorotatory isomers of praeruptorin A and praeruptorin B are naturally less abundant than their dextrorotatory enantiomers [6], indicating a chiral preference in the herb. In addition to praeruptorin A and praeruptorin B, there are several other pyranocoumarin constituents naturally present as mixtures of enantiomers in the herb, but so far, only (±)-praeruptorin A (**2**, Figure 1) has been enantioseparated by reversed-phase and normal-phase chiral chromatography [13,14].

At present, the absolute configurations of this type of coumarin compounds are usually determined by acidic or alkaline hydrolysis in combination of gel column chromatographic separation [6,7,15]. However, the method using acid or base hydrolysis requires large amounts of pure compounds and the absolute configuration might be altered during acid or alkaline treatment [7,15]. Circular dichroism (CD) has also been adopted [13,16], but the CD spectrum couldn’t distinguish the enantiomerically enriched compound from its pure enatiomer [17]. In contrast, enzyme-mediated hydrolysis generally breaks the ester bond without affecting the absolute configuration [18,19]. Our previous study on metabolism of (±)-praeruptorin A by hepatic proteins from humans and rats also revealed that *cis*-khellactone obtained from the levorotatory enantiomer of praeruptorin A or its antipode retained the same absolute configuration as the parent enantiomer [14], indicating that the absolute configuration of pyranocoumarin constituents could be determined from their hydrolytic products formed from enzyme-mediated reactions. On the other hand, chiral LC-MS/MS analysis affords high selectivity and sensitivity to allow the determination of drugs and metabolites at low concentrations, thus, has been widely adopted in bioanalysis.

Therefore, in the present study, a method that combines enzymatic hydrolysis followed by chiral LC-MS/MS analysis of the metabolites formed was adopted for the first time to determine the absolute configurations of angular-type pyranocoumarin compounds from Qian-hu. The results obtained were consolidated with data from polarimetric analysis and NMR data or by comparison with the data of the reference compounds.

## 2. Results and Discussion

### 2.1. Identification of Pyranocoumarins from Qian-hu

By comparing the NMR, MS data and optical rotations with the literature values (Table 1 and Table 2), compounds **1**–**11** (Figure 1) were identified as (±)-*cis*-khellactone (**1**), (±)-praeruptorin A [(±)*cis-*3′-angeloyl-4′-acetylkhellactone, **2**], praeruptorin B [*cis*-3′,4′-diangeloylkhellactone, **3**], (+)-praeruptorin E [(+)-(3′*S*,4′*S*)-3′-angeloyl-4′-isovalerylkellactone, **4**], *cis*-3′-acetyl-4′-angeloylkhellactone (**5**), *cis*-3′-isovaleryl-4′-acetylkhellactone (**6**), (+)-*cis*-(3′*S*,4′*S*)-3′-angeloyl-4′-senecioylkhellactone (**7**), (+)-*cis*-(3′*S*,4′*S*)-3′,4′-diisovalerylkhellactone (**8**), (−)-*cis*-(3′*R*,4′*R*)-3′-angeloylkhellactone (**9**), (−)-*cis*-(3′*R*,4′*R*)-4′-angeloylkhellactone (**10**) and (+)-*trans*-(3′*S*,4′*R*)-3′-angeloylkhellactone (**11**), respectively.

### 2.2. Enantioseparation of Mixtures of Angular-Type Pyranocoumarins

**Figure 2:**
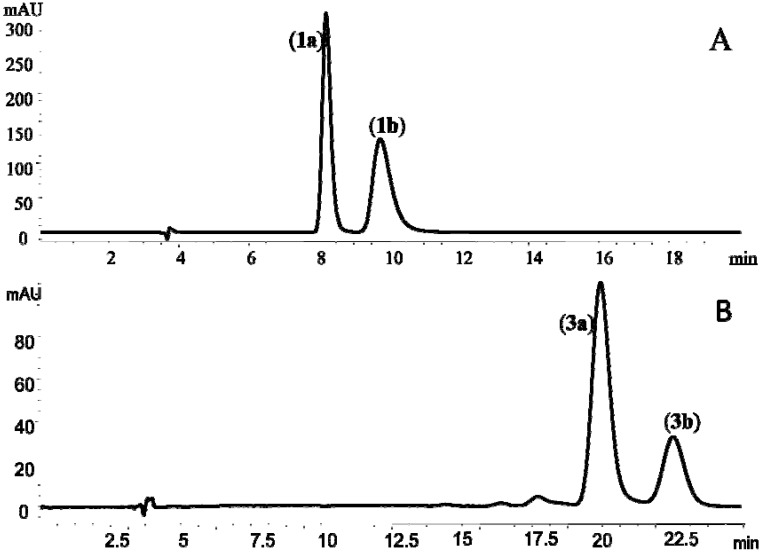
Typical HPLC-UV (323 nm) chromatograms of compounds **1** (A) and **3** (B) on a CHIRALPAK AD-RH semi-preparative column.

Under the present analytical conditions, the enantiomers of (±)-*cis*-khellactone (**1**) achieved good separation on a CHIRALPAK AD-RH semi-preparative column (Figure 2A). The two compounds were eluted at the retention times of 6.7 min (**1a**) and 9.7 min (**1b**), respectively. Their optical rotations were 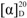 +57° (*c*, 1.0) and 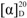 −55° (*c*, 1.0), which were in good agreement with those reported previously for (+)-*cis*-khellactone and (−)-*cis*-khellactone, respectively [20]. Thus, **1a** was assigned as (+)-*cis*-(3′*S*,4′*S*)-khellactone and **1b** as (−)-*cis*-(3′*R*,4′*R*)-khellactone (Figure 1 and Figure 2A).

Enantioseparation of (±)-praeruptorin B (*cis*-3′,4′-diangeloylkhellactone, **3**) was achieved on the same chiral semi-preparative column using a mobile phase with a different acetonitrile/H_2_O composition. The two enantiomers were eluted at 19.8 min (**3a**) and 22.9 min (**3b**), respectively. The optical rotations measured [3a, 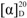 +37° (*c*, 1.0); **3b**, 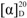 −36° (*c*, 1.0)] for the enantiomers were similar to the data reported by Okuyama and co-workers [7]. Thus the two enantiomers were identified as (+)-praeruptorin B [(+)-*cis*-(3′*S*,4′*S*)-3′,4′-diangeloylkhellactone, **3a**] and (−)-praeruptoin B [(−)-*cis*-(3′*R*, 4′*R*)-3′,4′-diangeloylkhellactone, **3b**] (Figure 1 and Figure 2B).

### 2.3. Determination of Absolute Configuration of Angular-Type Pyranocoumarins

Our previous studies [14,21] and Ruan’s report [22] on the metabolisms of (±)-praeruptorin A (**2**), (+)-praeruptorin A (**2a**), (−)-praeruptorin A (**2b**), (+)-praeruptorin B (**3a**) and (+)-praeruptorin E (**4**) revealed that all these angular-type pyranocoumarins underwent stepwise hydrolysis and generated *cis*-khellactone without alteration of the absolute configuration when they were incubated with liver microsomal proteins from rats or humans in the presence of a NADPH-regenerating system. This finding suggests that the absolute configurations of this type of coumarins can be determined from the configurations of C-3′ and C-4′ of khellactone produced from hydrolysis of the parent coumarins by rat or human hepatic phase I isozymes. 

**Table 1:** HPLC-MS/MS data and optical rotations of pyranocoumarins from Qian-hu and their hydrolytic products.

compound	MS^1^	MW	MS^2^	Optical rotation ( *c* 1.0, CDCl_3_)	Hydrolytic metabolite(+/− *cis*-khellactone)	Identity
**1**	263[M+H]^+^; 285[M+Na]^+^	262	245,203	0°	+:− = 1:1	(±)- *cis*-khellactone
**1a**	263[M+H]^+^; 285[M+Na]^+^	262	245,203	−55°	-	(−)- *cis*-(3′*S*, 4′*S*)-khellactone
**1b**	263[M+H]^+^; 285[M+Na]^+^	262	245,203	+57°	+	(+)- *cis*-(3′*R*, 4′*R*)-khellactone
**2**	404[M+NH_4_]^+^; 409[M+Na]^+^	386	327,245,227	0°	+:− = 1:1	(±)- *cis*-3′-angeloyl-4′-acetylkhellactone
**2a**	404[M+NH_4_]^+^; 409[M+Na]^+^	386	327,245,227	+59°	-	(+)- *cis*-(3′*S*, 4′*S*)-3′-angeloyl-4′-acetylkhellactone
**2b**	404[M+NH_4_]^+^; 409[M+Na]^+^	386	327,245,227	−61°	+	(−)- *cis*-(3′*R*, 4′*R*)-3′-angeloyl-4′-acetylkhellactone
**3**	444[M+NH_4_]^+^; 449[M+Na]^+^	426	327,245,227	+3.4°	+:− = 2:3	*cis*-3′, 4′-diangeloxylkhellactone
**3a**	444[M+NH_4_]^+^; 449[M+Na]^+^	426	327,245,227	+36°	-	(+)- *cis*-(3′*S*, 4′*S*)-3′, 4′-diangeloxylkhellactone
**3b**	444[M+NH_4_]^+^; 449[M+Na]^+^	426	327,245,227	−37°	+	(−)- *cis*-(3′*R*, 4′*R*)-3′, 4′-diangeloxylkhellactone
**4**	446[M+NH_4_]^+^; 451[M+Na]^+^	428	327,245,227	+35°	-	(+)- *cis*-(3′*S*, 4′*S*)-3′-angeloxyl-4′-isovalerylkhellactone
**5**	404[M+NH_4_]^+^; 409[M+Na]^+^	386	309,245,227	+2.9°	+:− = 7:1	*cis*-3′-acetyl-4′-angeloylkhellactone
**5a**	404[M+NH_4_]^+^; 409[M+Na]^+^	386	309,245,227	+3.5°	+	(+)- *cis*-(3′*R*, 4′*R*)-3′-acetyl-4′-angeloylkhellactone
**6**	406[M+NH_4_]^+^; 411[M+Na]^+^	388	329,245,227	+27°	+:− = 1:8	*cis*-3′-isovaleryl,4′-acetylkhellactone
**6a**	406[M+NH_4_]^+^; 411[M+Na]^+^	388	329,245,227	+33°	-	(+)- *cis*-(3′*S*, 4′*S*)-3′-isovaleryl,4′-acetylkhellactone
**7**	444[M+NH_4_]^+^; 449[M+Na]^+^	426	327,245,227	+31°	-	(+)- *cis*-(3′*S*, 4′*S*)-3′-angeloyl-4′-senecioylkhellactone
**8**	448[M+NH_4_]^+^; 453[M+Na]^+^	430	329,245,227	+35°	-	(+)- *cis*-(3′*S*, 4′*S*)-3′,4′-diisovalerylkhellactone
**9**	367[M+Na]^+^; 383[M+K]^+^	344	267,245,227	−57°	+	(−)- *cis*-(3′*R*, 4′*R*)-3′-angeloylkhellactone
**10**	367[M+Na]^+^; 383[M+K]^+^	344	267,245,227	−39°	+	(−)- *cis*-(3′*R*, 4′*R*)-4′-angeloylkhellactone
**11**	367[M+Na]^+^; 383[M+K]^+^	344	327,245,227	+11°	N.A.	(+)- *trans*-(3′*S*, 4′*R*)-3′-angeloylkhellactone

N.A.: not applicable.

**Table 2:** ^1^H-NMR spectroscopic data of angular-type pyranocoumarin compounds **1**-**11** in CDCl_3_ (600 M).

Position	Compound										
	1	2	3	4	5	6	7	8	9	10	11
3	6.27 (d, 9.5)	6.26 (d, 9.5)	6.23 (d, 9.5)	6.25 (d, 9.6)	6.24 (d, 9.6)	6.25 (d, 9.6)	6.23 (d, 9.5)	6.24 (d, 9.5)	6.27 (d, 9.6)	6.24 (d, 9.5)	6.27 (d, 9.5)
4	7.67 (d, 9.5)	7.61 (d, 9.5)	7.60 (d, 9.5)	7.60 (d, 9.6)	7.60 (d, 9.6)	7.61 (d, 9.6)	7.59 (d, 9.5)	7.60 (d, 9.5)	7.65 (d, 9.6)	7.61 (d, 9.5)	7.67 (d, 9.5)
6	7.34 (d, 8.7)	7.37 (d, 8.6)	7.37 (d, 8.6)	7.35 (d, 8.6)	7.37 (d, 8.6)	7.36 (d, 8.6)	7.36 (d, 8.6)	7.36 (d, 8.6)	7.36 (d, 8.5)	7.38 (d, 8.7)	7.35 (d, 8.6)
7	6.81 (d, 8.7)	6.82 (d, 8.6)	6.82 (d, 8.6)	6.81 (d, 8.6)	6.82 (d, 8.6)	6.81 (d, 8.6)	6.81 (d, 8.6)	6.81 (d, 8.6)	6.81 (d, 8.5)	6.82 (d, 8.7)	6.83 (d, 8.6)
3′	3.89 (d, 5.0)	5.37 (d, 5.0)	5.46 (d, 5.0)	5.41 (d, 4.8)	5.37 (d, 4.8)	5.34 (d, 5.0)	5.42 (d, 4.8)	5.34 (d, 4.8)	5.49 (d, 4.8)	4.10 (d, 4.8)	5.28 (d, 3.6)
4′	5.23 (d, 5.0)	6.65 (d, 5.0)	6.72 (d, 5.0)	6.63 (d, 4.8)	6.65 (d, 4.8)	6.57 (d, 5.0)	6.67 (d, 4.8)	6.57 (d, 4.8)	5.25 (d, 4.8)	6.51 (d, 4.8)	5.09 (d, 3.6)
5′	1.42 (s)	1.45 (s)	1.47 (s)	1.46 (s)	1.45 (s)	1.42 (s)	1.46 (s)	1.45 (s)	1.45 (s)	1.45 (s)	1.42 (s)
6′	1.48 (s)	1.49 (s)	1.51 (s)	1.49 (s)	1.48 (s)	1.46 (s)	1.50 (s)	1.47 (s)	1.51 (s)	1.50 (s)	1.52 (s)
2′′	-	-	-	-	-	2.26 (m); 2.25 (m)	-	2.30 (m); 2.20 (m)	-	-	-
3′′	-	6.14 (q, 7.2)	6.13 (q, 7.2)	6.13 (q, 7.2)	2.11 (s)	2.10 (m)	6.13 (q, 7.2)	2.15 (m)	6.18 (q, 7.2)	-	6.11 (q, 7.2)
4′′	-	1.97 (d, 7.2)	2.00 (d, 7.2)	1.98 (d, 7.2)	-	0.98 (d, 7.2)	1.98 (d, 7.2)	0.98 (d, 7.2)	2.00 (d, 7.2)	-	1.92 (d, 7.2)
5′′	-	1.88 (s)	1.87 (s)	1.89 (s)	-	0.98 (d, 7.2)	1.86 (s)	0.98 (d, 7.2)	1.91 (s)	-	1.85 (s)
2′′′	-	2.12 (s)	-	2.28 (m); 2.20 (m)	-	2.15 (s)	5.63 (s)	2.30 (m); 2.20 (m)	-	-	-
3′′′	-	-	7.25 (q, 7.2)	2.14 (m)	7.26 (q, 7.2)	-	-	2.15 (m)	-	6.13 q, 7.2	-
4′′′	-	-	1.98 (d, 7.2)	0.97 (d, 7.2)	2.01 (d, 7.2)	-	1.89 (s)	0.98, d, 7.2	-	2.02, d, 7.2	-
5′′′	-	-	1.85 (s)	0.97 (d, 7.2)	1.88 (s)	-	2.20 (s)	0.98, d, 7.2	-	1.91, s	-
Ref.	[6]	[6]	[6]	[8]	[23]	[24,25]	[26]	[26,27]	[14]	[14]	[20]

To confirm this speculation, (+)-praeruptorin A [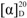 +59° (*c*, 1.0), **2a**], (−)-praeruptorin A [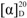 −61° (*c*, 1.0), **2b**], (+)-praeruptorin E (**4**), and the two metabolites of **2b** formed in rat plasma, (−)-*cis*-(3′*R*,4′*R*)-3′-angeloyl khellactone [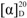 −57° (*c*, 1.0), **9**] and (−)-*cis*-(3′*R*,4′*R*)-4′-angeloyl-khellactone [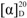 −39° (*c*, 1.0), **10**], were incubated with rat liver microsomes. The chemical structures and absolute configurations of **2a**, **2b**, **9** and **10** have been unambiguously identified using NMR analysis and optical rotation data in our laboratory [14], while **4** was commercially available and its chemical structure was double checked by comparing its mass spectral profile and optical rotation data [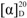 +35° (*c*, 1.0)] with the literature report [8].

**Figure 3:**
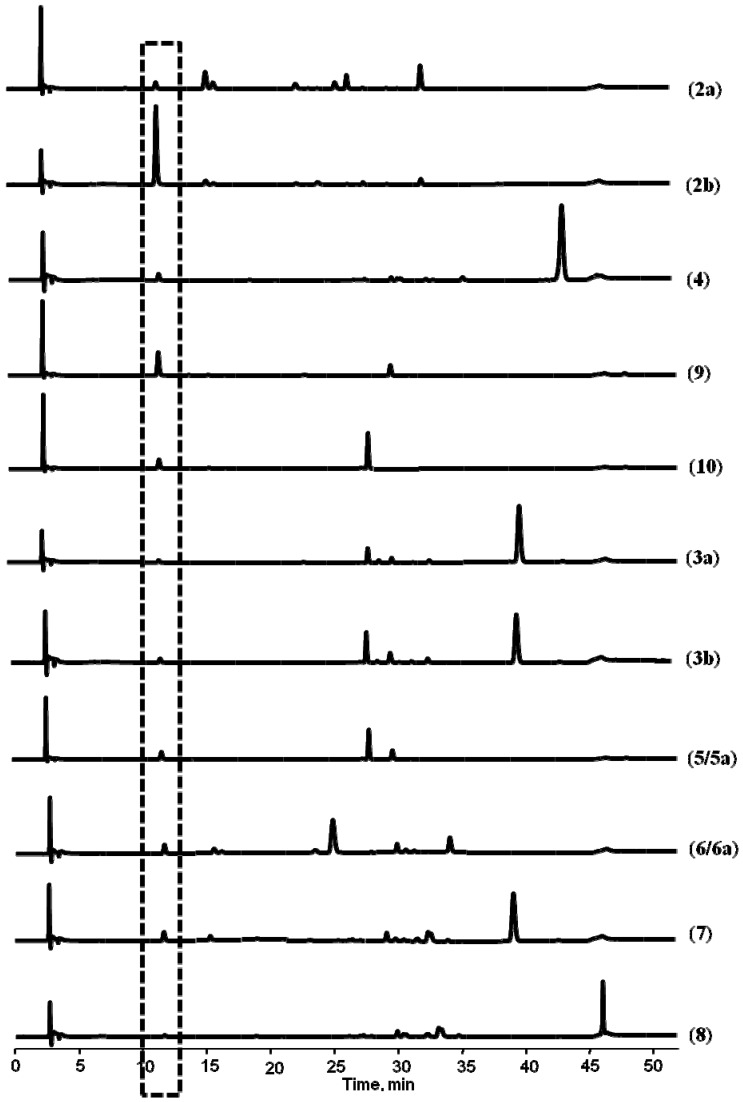
Typical achiral HPLC-UV (323 nm) chromatograms of incubations of compounds**2–10** in rat liver microsomes in the presence of a NADPH-regenerating system.

**Figure 4:**
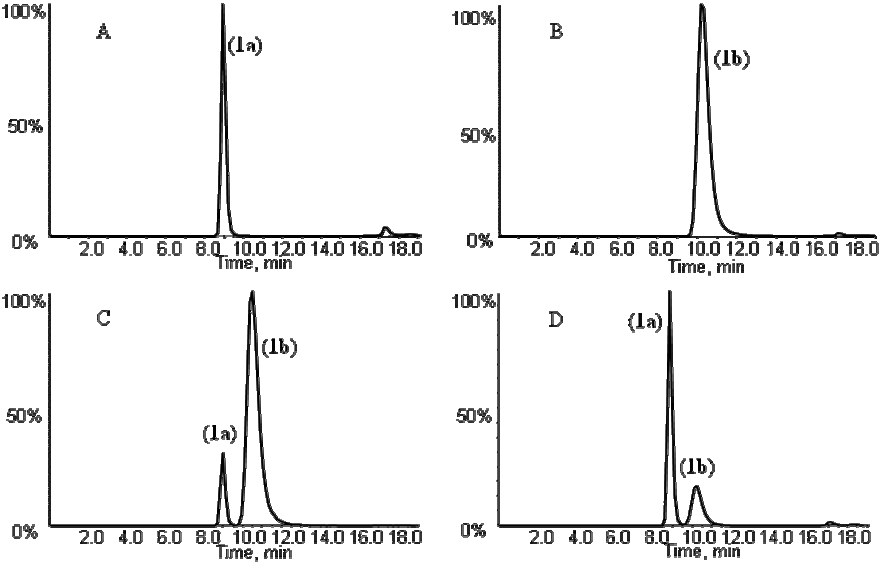
Typical extracted ion current chromatograms (XIC) (263.1 > 203.1) of authentic **1a** (A) and **1b** (B), and samples from incubations of compound **5** (C) and **6** (D) with rat liver microsomes under chiral LC-MS/MS analytical conditions.

When incubated with rat liver microsomes (RLMs) in presence of the NADPH-regenerating system, all these five pyranocoumarins generated *cis*-khellactone which was eluted at 12.0 min under present achiral HPLC conditions and exhibited identical UV and MS spectra to those of the authentic (±)-*cis*-khellactone (Figure 3). When the *cis*-khellactone produced from each reaction was collected and analyzed using chiral LC-MS/MS, the products formed from (+)-praeruptorin E (**4**) and (+)-praeruptorin A (**2a**) exhibited the same retention time at 9.0 min as that of (−)-*cis*-khellactone (**1b**, Figure 4A), while those metabolites of (−)-praeruptorin A (**2b**), (−)-(3′*R*, 4′*R*)-3′-angeloylkhellactone (**9**) and (−)-(3′*R*,4′*R*)-4′-angeloylkhellactone (**10**) shared the same retention time (10.6 min) with (+)-*cis*-khellactone (**1a**, Figure 4B).

These results together with the optical rotation data of these parent pyranocoumarins (Table 1) support that their respective hydrolytic metabolite *cis*-khellactone keeps the absolute configurations during hydrolysis of the parent compounds in rat liver microsomal proteins. 

In contrast, when **2a** [(+)-*cis*-(3′*S*,4′*S*)-3′-angeloyl-4′-acetylkhellactone] was subjected to basic hydrolysis [20], the formation of compound **11** [(+)-*trans*-(3′*S*,4′*R*)-3′-angeloylkhellactone] was evidenced by NMR and LC-MS/MS analysis and its optical rotation (Table 1 and Table 2). Peucedanocoumarins I, II and III, which are also *trans*-khellactone type coumarins, yielded *cis*-khellactone after alkaline hydrolysis [23]. Thus, epimerization might occur during alkaline treatment of pyranocoumarins and finally may cause misidentification of the absolute configurations of parent compounds.

This method was further applied to the determination of the absolute configuration of another four pyranocoumarin compounds **5a**, **6a**, **7** and **8** isolated from Qian-hu. *cis*-Khellactone obtained from the enzymatic hydrolysis of compound **5a** was eluted at 12.0 min on the achiral column (Figure 3) and only appeared at 10.6 min on the chiral LC-MS/MS (Figure 4B), and identified as (+)-*cis*-khellactone by comparison with the reference compound. Thus a (3′*R*,4′*R*) configuration of compound **5a** was suggested. Furthermore, the optical rotation of **5a**in chloroform was 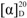 +3.5° (*c*, 1.0) and the NMR data supported the structure as *cis*-3′-acetyl-4′-angeloylkhellactone (Table 2). These findings demonstrated that the absolute configuration of **5a** predicted from the absolute configuration of its metabolite *cis*-khellactone corresponded well with the structure identified based on LC-MS/MS, NMR and polarimetric analysis (Table 1 and Table 2). These data of **5a** were in good agreement with those of pteryxin [(3′*R*, 4′*R*)-3′-acetyl-4′-angeloylkhellactone], which has been isolated from *P. praeruptorum* [23], thus, compound **5a** was unambiguously identified as pteryxin [(+)-*cis*-(3′*R*,4′*R*)-3′-acetyl-4′-angeloyl-khellactone].

Similarly, the end products obtained from hydrolysis of compounds **6a**, **7**, **8** by RLMs (Figure 3) showed the same retention time as that of (-)-*cis*-khellactone on the chiral LC-MS/MS system (Figure 4A), supporting the existence of *cis*-(3′*S*,4′*S*) configurations in these compounds. Correspondingly, the NMR data (Table 2) of compound **6a** supported it as *cis*-3′-isovaleryl-4′-acetyl khellactone and polarimetric measurement revealed a positive optical rotation [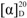 +33° (*c*, 1.0)]. Compound **6a** was thereby identified as (+)-*cis*-(3′*S*,4′*S*)-3′-isovaleryl-4′-acetylkhellactone. NMR analysis (Table 2) of compounds **7** and **8** identified their structures as *cis*-3′-angeloyl-4′-senecioylkhellactoneand *cis*-3′,4′-diisovalerylkhellactone, respectively. Their optical rotations were +31° (*c*, 1.0) and +35° (*c*, 1.0). Taken together, the structures were assigned as (+)-*cis*-(3′*S*,4′*S*)-3′-angeloyl-4′-senecioylkhellactone (**7**) and (+)-*cis*-(3′*S*, 4′*S*)-3′,4′-diisovalerylkhellactone (**8**). So far, (±)-dihydrosamidin, the racemic form of compound **6a**, has been isolated from *P. Turgeniifolium* [24,25], while neither the racemate nor any of the single enantiomers has been reported for Qian-hu. Both 3′-angeloyl-4′-senecioylkhellactone and 3′,4′-diisovalerylkhellactone have been reported in *P. japonicum*,but not in *P. praeruptorum *(Qian-hu) [26] and their absolute configurations were not determined in the previous report [27]. Again, determination of the absolute configurations of pyranocoumarin compounds **5a**, **6a**, **7** and **8** based on chiral LC-MS/MS analysis of their respective hydrolytic metabolite *cis*-khellactone formed by RLMs were proved to be feasible and should be applicable to other pyranocoumarins that have a hidden C-3' and/or C-4' hydroxy functionality that can be released by enzymatic transformations.

It’s interesting to note that compounds **5** and **6**, which were obtained from Qian-hu to prepare **5a** and **6a** through crystallization, exhibited similar positive optical rotation and identical NMR data as compounds **5a** and **6a**, respectively. However, when subjected to enzymatic hydrolysis, both compounds formed the two optical isomers of *cis*-khellactone (Figure 4C,D), indicating that compounds **5** and **6** were isolated from Qian-hu as enantiomerically enriched compounds. This was further confirmed by two peaks observed in chromatograms obtained from enantioseperation of compounds **5** and **6** (data not shown) and the enantiometric ratios (*±*) of *cis*-khellactone formed from **5** (7/1) and **6** (1/8) by RLMs (Table 1). Thus, the NMR data plus net optical rotation could not distinguish the enantiomerically enriched angular-type pyranocoumarin from its pure enantiomer.

In the present study, (±)-praeruptorin B (**3**) was enantioseparated, for the first time, to obtain the two enantiomers. The two optical isomers (**3a** and **3b**) were also subjected to enzymatic hydrolysis followed by chiral LC-MS/MS analysis individually. Similar to those observed with the two isomers from (±)-praeruptorin A (**2**), *cis*-khellactone from (+)-praeruptorin B (**3a**) shared the same retention time (9.0 min) with (−)-*cis*-khellactone (Figure 4A), while *cis*-khellactone from (−)-praeruptorin B (**3b**) was observed at 10.6 min, the same as (+)-*cis*-khellactone (Figure 4B), suggesting that the metabolite *cis*-khellactone keep the absolute configuration as their respective parent optical isomer. Again, this result agreed well with that obtained from a combination of NMR and polarimetric analysis (Table 1 and Table 2) in the present study and the previous report [7]. Thus, (+)-praeruptorin B (**3a**) was identified as (+)-*cis*-(3′*S*,4′*S*)-3′,4′-diangeloylkhellactone and (−)-praeruptorin B (**3b**) as (−)-*cis*-(3′*R*,4′*R*)-3′,4′-diangeloylkhellactone.

The stereoselectivity in pharmacological actions [13], metabolism [14] and absorption [28] of praeruptorin A have been revealed by previous studies, indicating that it is of great importance to determine the absolute configurations of the angular-type pyranocoumarins when correlate their structures with the resultant biological interactions during pharmacological/toxicological evaluation and/or ADME screening. Furthermore, because most pyranocoumarins from Qian-hu naturally exist as enantiomers and/or diastereoisomers, misidentification of the identity of the compound studied has occurred [29,30,31]. Thus, it is crucial to establish a method which enables rapid determination of the absolute configurations of the pyranocoumarin compounds to be worked on. These findings obtained in the present study demonstrated that it is feasible to determine the absolute configurations of pyranocoumarins from that of the hydrolytic product khellactone using enzymatic hydrolysis coupled with chiral LC-MS/MS analysis. Because of the high sensitivity and selectivity afforded by LC-MS/MS techniques, this method can be used to determine the configuration of compounds of this chemical type with advantages in lower quantity of the tested compounds required for *in vitro* incubation.

## 3. Experimental

### 3.1. Materials

Qian-hu was obtained from Ningguo, Anhui Province, China and the crude drug was authenticated as the dried roots of *P. praeruptorum* DUNN by Professor Pengfei Tu from Department of Natural Medicines, Peking University (Beijing, China). The specimens were stored at the State Key Laboratory of Quality Research in Chinese Medicine, University of Macau.

The crude drug (6 kg) was crushed into a powder and extracted with 95% ethanol (50 L × 3, 80 °C) to afford an extract (1.83 kg). An aliquot of the extract (50 g) was loaded onto a silica gel column (200–300 mesh, 800 g) and fractionated with petroleum ether-ethyl acetate (20:1→1:2, *v*/*v*) to get 11 fractions (Fractions 1–11). Fraction 4 (4.2 g) was loaded onto another silica gel column (200–300 mesh, 100 g) was and compound **2** (432 mg) was eluted with petroleum ether-ethyl acetate (8:1→1:1, *v*/*v*). Compounds **3** (195 mg) and **4** (52 mg) were obtained by recrystallization of Fraction 5 (5.3 g) and Fraction 8 (7.1 g) from MeOH, respectively. Compound **6** (12 mg) was isolated from the remaining solution of Fraction 5 using a semi-preparative COSMOSIL 5C18-AR-II column (250 mm × 10.0 mm i.d., particle size 5 µm, Nacalai Tesque, Kyoto, Japan) that eluted with MeOH–H_2_O (65:35, *v*/*v*) at a flow rate of 2.5 mL/min. The remaining solution of Fraction 8 was introduced to the same semi-preparative HPLC system and eluted with MeOH-H_2_O (69:31, *v*/*v*) to afford **7** (11 mg) and **8** (17 mg). Compound **5** (13 mg) was prepared from Fraction 3 by semi-preparative HPLC with an isocratic elution utilizing MeOH-H_2_O (77:23, *v*/*v*). Compounds **5a** [(+)-*cis*-(3′*R*,4′*R*)-3′-acetyl-4′-angeloyl-khellactone] and **6a** [(+)-*cis*-(3′*S*,4′*S*)-3′-isovaleryl-4′-acetylkhellactone] were obtained as white needles by recrystallization of **5** and **6 **from methanol.

A basic hydrolysis of **2** (70 mg) was carried out to obtain **1** (23 mg) following a procedure described previously by our group [14]. (+)-Praeruptorin A [(+)-*cis*-(3′*S*,4′*S*)-3′-angeloyl-4′-acetyl- khellactone, **2a**] and (−)-praeruptorin A [(−)-*cis*-(3′*R*,4′*R*)-3′-angeloyl-4′-acetylkhellactone, **2b**] were obtained by enantioseperation of compound **2** according to our previous report [14]. (−)-*cis*-(3′*R*,4′*R*)-3′-angeloylkhellactone (**9**) and (−)-*cis*-(3′*R*,4′*R*)-4′-angeloylkhellactone (**10**) were prepared from an incubation of compound **2b** with rat plasma and unambiguously identified using NMR and LC-MS/MS analysis [14]. Compound **11** was obtained from basic hydrolysis of compound **2a**, according to a method reported by Wu and co-workers [20]. Additionally, authentic compounds **2**, **2a** and **4** were purchased from Shanghai Traditional Chinese Medicine Research Center (Shanghai, China) and their identities were confirmed using LC-MS/MS and polarimetric analysis. The purity of all the compounds was all above 98% as determined by achiral HPLC-UV. The identities of all pyranocoumarin constituents **1**–**11** isolated from Qian-hu were identified using polarimetric analsyis, LC-UV-MS/MS and NMR analysis and confirmed by comparison with previous reports. The spectrometric and spectroscopic data were summarized in Table 1 and Table 2.

Pooled rat liver microsomes (RLMs) were prepared at the School of Biomedical Sciences, the Chinese University of Hong Kong by differential centrifugation according to a standard procedure reported previously [32]. The content of microsomal proteins was determined using Lowry’s method [33] and stored at −80 °C until use.

Glucose 6-phosphate (G-6-P), glucose-6-phosphate dehydrogenase (G-6-PD), and *β*-nicotinamide adenine dinucleotide phosphate (*β*-NADP^+^) were obtained from Sigma-Aldrich Corp. (St. Louis, MO, USA). Formic acid, acetonitrile and methanol of HPLC grade were purchased from Merck (Darmstadt. Germany). Ultra-pure water was obtained in house using a Milli-Q plus water purification system (Millipore, Bedford, MA, USA).

### 3.2. Enantioseparation of Angular-type Pyranocoumarins from Qian-hu

Enantioseparation of the enantiometric mixtures (±)-*cis-*khellactone (**1**) and (±)-praeruptorin B (**3**) were carried out on a semi-preparative CHIRALPAK AD-RH column (10 × 250 mm I.D., particle size 5 µm, Daicel, Tokyo, Japan). The mobile phases were acetonitrile-H_2_O with a volumetric ratio of 30/70 for **1** and 45/55 for **3**. The flow rates were 2.5 mL/min for elution of both analytes. Each analyte eluted was collected separately and the solvent was removed under reduced pressure to yield **1a** (9.5 mg) and **1b** (8.5 mg), **3a** (23.6 mg) and **3b** (19.9 mg). The purity of all the enantiomers was more than 98% as determined using chiral LC-UV/MS/MS.

### 3.3. Polarimetric and NMR Analysis

The polarimetric analysis was performed on a Perkin-Elmer 243B digital polarimeter (PerkinElmer, Netherlands) in chloroform at 589.3 nm, 20 °C. Compounds **1**–**11** were dissolved in CDCl_3_ containing 0.3% trimethylsilane (TMS) and their ^1^H NMR spectra were acquired on a Bruker Avance 600 spectrometer (600 MHz, Bruker GmbH, Bremen, Germany). Chemical shifts were reported in *δ* scale in units of part per million (ppm) with TMS as the reference, and coupling constants (*J*) were expressed in units of Hertz (Hz). 

### 3.4. Hydrolysis of Angular-type Pyranocoumarins from Qian-hu by Rat Liver Microsomes

Enzymatic hydrolysis of each of the angular-type pyranocoumarins (final concentration 25 µM) was carried out in a 200 µL reaction system containing 1 mg/mL of rat liver microsomal proteins in 0.1 M potassium phosphate buffer (pH 7.4) at 37 °C. The reactions were initiated by adding the NADPH-regenerating system (4 mM MgCl_2_, 1 mM *β*-NADP^+^, 1 mM G-6-P and 1 U/mL of G-6-PD). Reactions were stopped at 60 min by adding an equal volume of ice-cold methanol and the samples centrifuged to remove the proteins before subjected to achiral HPLC-UV analysis. 

### 3.5. Achiral HPLC-UV Analysis

The achiral HPLC-UV analysis was performed on an Agilent 1200 series liquid chromatographic system (Agilent Technologies, Palo Alto, CA, USA) that equipped with a vacuum degasser, a quaternary pump, an autosampler and a diode array detector (DAD) system. Data acquisition was controlled by an Agilent ChemStation B 3.0 software. Sample separation was performed on an ODS reversed-phase C_18_ column (250 mm × 4.6 mm I.D., particle size 5 µm, Agilent) at 35 °C. The mobile phase consisted of water containing 0.1% formic acid (A) and methanol (B). A gradient elution was carried out at 1.0 mL/min as follows: 0–4 min, 25–50% B; 4–16 min, 50–53% B; 16–17 min, 53–56% B; 17–20 min, 56% B; 20–23 min, 56–69% B; 23–40 min, 69–76% B; 40–41 min, 76–100% B; 41–52 min, 100% B. All reference compounds and samples from reactions were monitored at 323 nm and UV spectra were recorded over 200–400 nm. The injection volume was 70 µL. *cis*-Khellactone, the end product yielded from the aforementioned reactions of all pyranocoumarins, was eluted at the retention time of 12.0 min under the present analytical conditions and collected separately for further chiral LC-MS/MS analysis.

### 3.6. Chiral HPLC-MS/MS Analysis

Chiral LC-MS/MS analysis was performed on an Agilent 1200SL series (Agilent) liquid chromatography coupled online with an API 4000 Q-trap^@^ mass spectrometer (Applied Biosystems, Foster City, CA, USA). The LC system consisted of a vacuum degasser, a binary pump, and an autosampler. Chromatographic separation was performed on an analytical CHIRALPAK AD-RH column (5 × 150 mm I.D., particle size 5 µm, Daicel, Tokyo, Japan), which was eluted with acetonitrile-H_2_O (30:70, v/v) at a flow rate of 0.65 mL/min. The mass spectrometer was equipped with a Turbo V^TM^ source and a Turbo Ion Spray probe (500 °C) and operated in positive ion multiple reactions monitoring (MRM) mode. Ion optics was tuned using polypropylene glycol (PPG) standard dilution solvents. Nitrogen was used as the nebulizer, heater, curtain and collision gases. Optimum ion source parameters for *cis-*khellactone were as follows: nebulizer (GS1), heater (GS2) and curtain gas flow rates 50, 50, 10 instrument units, respectively; ionspray voltage 4500 V; heater gas temperature 550 °C; declustering potential (DP) 100 V. The ion pairs used for monitoring *cis*-khellactone were 263.1 > 245.1 and 263.1 > 203.1. Collision energies (CE) were set as 25 eV and 30 eV for the two ion pairs, respectively. The Applied Biosystems Analyst Software package (Version 1.5) was used for instrument control, data acquisition and processing.

## 4. Conclusions

In the present study, a new method using enzymatic hydrolysis coupled with chiral LC-MS/MS analysis was developed to assist the determination of the absolute configuration of angular-type pyranocoumarins. It has been proved to be rapid and sensitive and demonstrated success in identification of this chemical type from Qian-hu. 
